# Machine Learning Prediction of Tongue Pressure in Elderly Patients with Head and Neck Tumor: A Cross-Sectional Study

**DOI:** 10.3390/jcm13082363

**Published:** 2024-04-18

**Authors:** Xuewei Han, Ziyi Bai, Kaoru Mogushi, Takeshi Hase, Katsuyuki Takeuchi, Yoritsugu Iida, Yuka I. Sumita, Noriyuki Wakabayashi

**Affiliations:** 1 Department of Advanced Prosthodontics, Graduate School, Medical and Dental Sciences, Tokyo Medical and Dental University, Tokyo 1138510, Japan; han.mfp@tmd.ac.jp (X.H.); baimfp@tmd.ac.jp (Z.B.); wakabayashi.rpro@tmd.ac.jp (N.W.); 2 Institute of Education, Tokyo Medical and Dental University, Tokyo 1138510, Japan; mogushi.mds@tmd.ac.jp (K.M.); 2121adm@tmd.ac.jp (T.H.);; 3 Faculty of Pharmacy, Keio University, Tokyo 1088345, Japan; 4 Center for Mathematical Modelling and Data Science, Osaka University, Osaka 5608531, Japan; 5 The Systems Biology Institute, Tokyo 1410022, Japan; 6 Department of Partial and Complete Denture, The Nippon Dental University School of Life Dentistry, Tokyo 1028159, Japan

**Keywords:** tongue pressure, head and neck tumor, machine learning, oral function, random forest, XGBoost, logistic regression, support vector machine

## Abstract

**Background:** This investigation sought to cross validate the predictors of tongue pressure recovery in elderly patients’ post-treatment for head and neck tumors, leveraging advanced machine learning techniques. **Methods:** By employing logistic regression, support vector regression, random forest, and extreme gradient boosting, the study analyzed an array of variables including patient demographics, surgery types, dental health status, and age, drawn from comprehensive medical records and direct tongue pressure assessments. **Results:** Among the models, logistic regression emerged as the most effective, demonstrating an accuracy of 0.630 [95% confidence interval (CI): 0.370–0.778], F1 score of 0.688 [95% confidence interval (CI): 0.435–0.853], precision of 0.611 [95% confidence interval (CI): 0.313–0.801], recall of 0.786 [95% confidence interval (CI): 0.413–0.938] and an area under the receiver operating characteristic curve of 0.626 [95% confidence interval (CI): 0.409–0.806]. This model distinctly highlighted the significance of glossectomy (*p* = 0.039), the presence of functional teeth (*p* = 0.043), and the patient’s age (*p* = 0.044) as pivotal factors influencing tongue pressure, setting the threshold for statistical significance at *p* < 0.05. **Conclusions:** The analysis underscored the critical role of glossectomy, the presence of functional natural teeth, and age as determinants of tongue pressure in logistics regression, with the presence of natural teeth and the tumor site located in the tongue consistently emerging as the key predictors across all computational models employed in this study.

## 1. Introduction

According to a survey in 2022, almost half of community-dwelling elderly adults in Japan have oral hypofunction [1]. The elderly who have undergone head and neck tumor resection are at greater risk of oral hypofunction, lower food intake diversity, or reduced masticatory performance, which can lead to malnutrition, weight loss, physical weakening (sarcopenia, dysphagia, oral frailty, etc.) and quality of life compared with the general elderly population [2,3,4,5,6,7,8,9,10]. Maximum tongue pressure (MTP) is both a key indicator of tongue function and one of the criteria for diagnosing oral hypofunction, according to the Japanese Society of Gerodontology (JSG), as well as a sensitive marker for swallowing dysfunction, malnutrition, dysphagia, and oral frailty [4,6,11,12]. Fujikawa et al. revealed that tongue pressure is a greater contributor to masticatory performance in denture wearers than in individuals with natural dentition, indicating the importance of maintaining sufficient tongue pressure [7].

It is regarded that MTP < 20 kPa is commonly found in cases of dysphagia or pneumonia-related mortality [5,8]. Additionally, Hasegawa et al. found that MTP < 15 kPa can be used as a criterion for dysphagia after surgery for head and neck cancer [10]. Previous studies have established that after the growth phase, tongue pressure decreases with advancing age; this tends to be greater in men than in women below the age of 60 years and is influenced by various factors including their nutritional status, sarcopenia, physical measurements (height and weight), total muscle mass, grip strength, aspects of mastication (e.g., chewing frequency and patterns), oral health (including the number of remaining teeth and dental treatment), and cognitive performance in individuals with either intact or incomplete dentition [6,13,14]. Fujikawa et al. and de Groot et al. found that MTP deteriorated after oral oncological treatment, and MTP was higher in individuals with more occlusal units, but the effects of defect configuration, defect size, and denture stability remain unclear [7,12]. However, given the complex anatomy and multifactorial nature of tongue pressure in patients with maxillofacial defects, it would be helpful to determine how surgical intervention, the type of reconstruction, remaining teeth, and denture rehabilitation can predict changes in tongue pressure so as to provide clinical guidance for multidisciplinary cooperation in the management of these patients.

To achieve the highest predictive performance, logistic regression (LR), support vector machine (SVM), random forest (RF), and extreme gradient boosting (XGB) were selected [15,16,17]. In recent years, machine learning techniques have been increasingly applied in the field of removable prosthodontics [18]. LR, SVM, RF, and XGB, which are recognized for their high accuracy, have been extensively used to cross validate risk factors associated with various diseases, underscoring their potential in enhancing diagnostic and predictive accuracies in clinical settings [19,20,21,22,23,24]. The RF and XGB algorithms are considered to have high sensitivity, specificity, and accuracy compared with traditional logistic regression models [16,17,19,20,21,22,23,24]. RF is a nonlinear algorithm that excels in handling both categorical and continuous variables without constraints, leveraging decision tree models to enhance prediction accuracy and maintain robustness in the presence of multicollinearity [16]. XGB implements gradient-boosted decision trees to make predictions and strengthens models that give weak predictions [19,20,21,22,23,24]. Previous studies of factors that affect MTP have employed multiple regression analysis in healthy elderly and younger adults, but rarely in patients with head and neck tumors [7,14,25]. RF has been applied to speech impairment [26], but a lack of studies has investigated the use of machine learning models to predict other oral functions, such as tongue pressure, in elderly patients with head and neck tumors.

In this study, we used LR, SVM, RF, and XGB with higher accuracy compared with conventional statistical analysis to develop predictive models for maximum tongue pressure (MTP) in elderly patients (aged 65 years or older) with head and neck tumors to cross validate risk factors of diminished tongue pressure. Our prediction models may provide actionable insights for prosthodontists to make an early diagnosis of tongue pressure problems. The models may also enable customized interventions based on patients’ features and may further improve quality of life by reducing the occurrence of dysphagia and aspiration resulting from decreased tongue pressure. The aim of this study was to construct machine learning models to cross validate factors that contribute to predicting tongue pressure patients with head and neck tumors. The null hypothesis is that there are no significant predictors among the factors analyzed in the present study that affect tongue pressure after treatment for head and neck tumors in patients aged 65 or older.

## 2. Materials and Methods

### 2.1. Patient Eligibility

Eighty patients who had undergone ablative surgery for head and neck tumors followed by rehabilitation with a dento-maxillary prosthesis at the dental hospital of our institution were enrolled. This study was approved by the Ethics Committee of Tokyo Medical and Dental University (approval no. D2022-004; 5 July 2022) and was conducted in accordance with the principles outlined in the Declaration of Helsinki and its subsequent amendments. Patient consent was obtained via the opt-out route, in which information about the research was presented in poster form at treatment locations.

### 2.2. Inclusion Criteria and Exclusion Criteria

The inclusion criteria were aged ≥65 years, with head and neck tumor resection, use of a well-fitting maxillary or mandible denture for at least 3 months, and completion of all dental treatment. The exclusion criteria were cognitive impairment, neurodegenerative disease affecting tongue movement, temporomandibular joint disorders, and unstable systemic diseases.

### 2.3. Study Design

Age, sex, number of present teeth that were defined as those in which crowns had erupted, and were excluded if they were not occluded, were residual roots, or were significantly mobile, occlusal units with and without denture, primary tumor site, and type of reconstruction (soft tissue reconstruction such as flap reconstruction or hard tissue reconstruction such as bone and/or metal plate reinforcement) were confirmed from medical records and intraoral examinations. The number of functional teeth was determined by considering both natural teeth and teeth that had been restored with crowns, replaced with a bridge or implants, and artificial teeth or removable dentures, but retained roots and third molars were not included in this count. Categorical variables are shown as frequencies or proportions, and continuous variables are expressed as the mean and standard deviation (SD) (Table 1).

**Figure 1:**
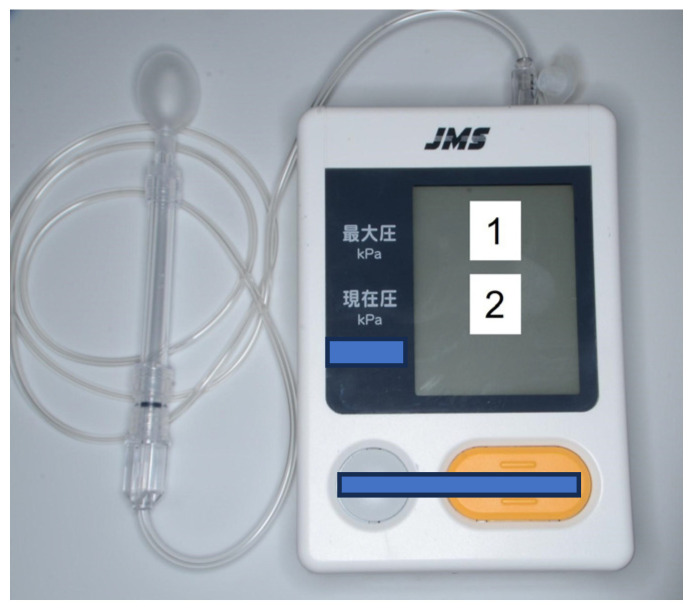
Tongue-pressure-measuring instrument equipped with a balloon probe. Maximum tongue pressure (kPa) is displayed in box 1, and current tongue pressure (kPa) is displayed in box 2.

A tongue-pressure-measuring instrument (TPM-02, JMS Co. Ltd., Hiroshima, Japan) (Figure 1) equipped with a balloon probe was used to measure MTP [4,5,6,27] (Figure 1). Patients with a maxillofacial prosthesis were instructed to sit straight, voluntarily elevate their tongue, and compress the inflated balloon three times between their tongue and the anterior part of the palate, which could include the denture base. The mean value was recorded [6,7]. The cutoff for tongue pressure was set at 20 kPa; Values of 20 kPa or higher were coded as “1” and values less than 20 kPa were coded as “0” [5,8].

### 2.4. Statistical Analysis

Predictive models for MTP employing machine learning techniques were developed, with LR and SVM implemented within the R environment (ver. 4.3.0) using the glm () function and the e1071 package, respectively. The analytical procedures were performed using R Studio (ver.4.3.1, Public Benefit Corporation, Boston, MA, USA), while training, analysis, and visualization of the RF and XGB models were performed in PyCharm (ver. 2023.2, JetBrains, Prague, Czech Republic) based on Python interpreter (Ver 3.11, Python Software Foundation, Wilmington, DE, USA), employing the scikit-learn and XGB libraries, respectively. The dataset was partitioned, with two-thirds of the data (*n* = 53) used for the training set and the remaining one-third (*n* = 27) for the testing set [21,28]. The significance level was set as α = 0.05. For SVM, RF, and XGB, the training process incorporated 5-fold cross-validation to fine-tune the hyperparameters of the classifiers [28]. For the RF model, the hyperparameters subjected to optimization included:

“max_depth”: 2,3,4,5,6,8,10,20
“min_samples_split”: 2,3,5
“n_estimators”: 10,20,30,50
“max_features”: ‘sqrt’, ‘log2’
“criterion”: “gini”, “entropy”

In the case of the XGB model, the parameters adjusted were:

“max_depth: 2,3,5,10
“booster”: ‘gbtree’,’gblinear’
“learning_rate”: 0.01,0.1,0.3,0.5
“n_estimators”: 10,20,30,50
“gamma”: 0,0.3,1.0
“reg_lambda”: 0,0.3,0.8,1
“reg_alpha”: 0,0.3,0.8,1
“silent”: 1.

To evaluate the performance of these models, metrics such as accuracy, F1 score, precision, recall, and the area under the receiver operating characteristic (AUC) were computed [15]. Within the SVM framework, the top five influential variables were identified using the recursive feature elimination technique [29]. Additionally, the feature importance scores in both the RF and XGB models were calculated, and the top five features with the highest scores were further analyzed to elucidate their predictive values and importance.

### 2.5. Evaluation of Sample Size

The appropriateness of the sample size was tested by the reference value of Rajput et al., i.e., they regarded sample size of a dataset as adequate when the dataset meets both two criteria (i) prediction accuracy > 80% and (ii) Cohen’s d > 0.5 [30].

## 3. Results

**Figure 2:**
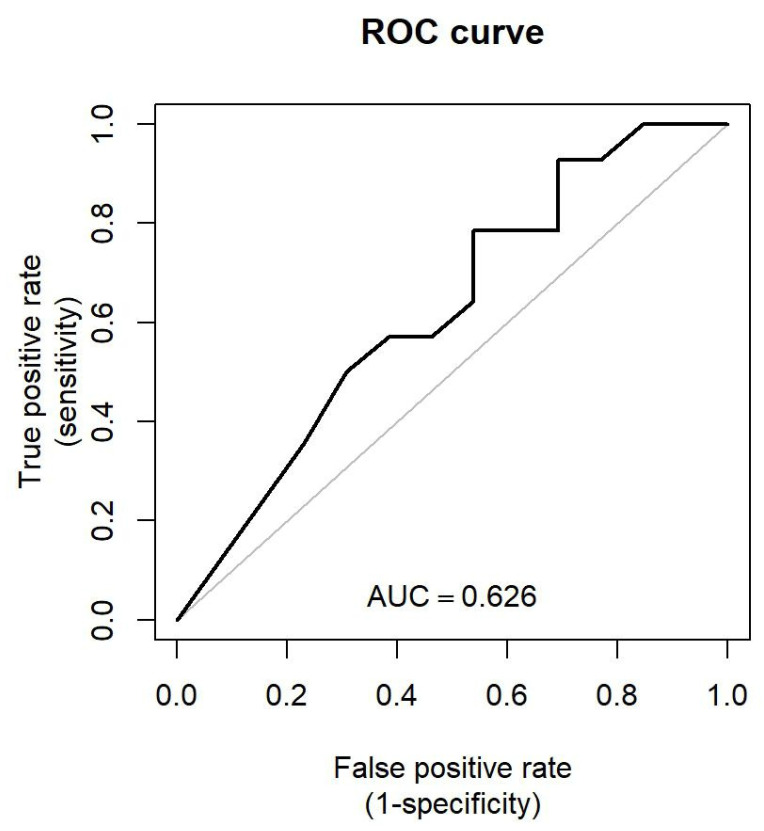
Receiver operating characteristic (ROC) curve of multiple logistic regression in testing dataset.

The patient characteristics and related variables are presented in Table 1. The model performance metrics are shown in Table 2. Based on the results of the analysis, we have sufficient evidence to reject the null hypothesis. In the LR model, glossectomy, *p* = 0.039 * (OR = 0.128, 95% CI: 0.018–0.898), functional teeth, *p* = 0.043 * (OR = 0.014, 95% CI: 0.000–0.882), and age, *p* = 0.044 * (OR = 5.335, 95% CI: 1.044–27.243) were identified as significant predictors of MTP (* *p* < 0.05) (Table 3). Among the four models evaluated, the LR model had the highest AUC of ROC at 0.626 [95% CI: 0.409–0.806] (Figure 2). In the SVM model, the top five predictors of MTP, identified using the recursive feature elimination technique, were occlusal units of natural teeth, tongue cancer, glossectomy, presence of teeth, and functional teeth, with an AUC of 0.582 [95% CI: 0.390-0.761]. For the RF model, the optimal parameters were identified as {‘clf__criterion’: ‘gini’, ‘clf__max_depth’: 5, ‘clf__max_features’: ‘sqrt’, ‘clf__min_samples_split’: 2, ‘clf__n_estimators’: 10}. The most influential features in the RF model were occlusal units (natural teeth) (0.178), present teeth (0.173), age (0.132), tongue cancer (0.088) and occlusal units with denture (0.081) with an AUC of 0.626 [95% CI: 0.385–0.843]. In the XGB model, the best parameters were {‘classifier__booster’: ‘gbtree’, ‘classifier__gamma’: 0.3, ‘classifier__learning_rate’: 0.01, ‘classifier__max_depth’: 3, ‘classifi-er__n_estimators’: 50, ‘classifier__reg_alpha’: 0, ‘classifier__reg_lambda’: 0, ‘classifier__silent’: 1}. The strongest predictors were occlusal units of natural teeth (0.395), followed by glossectomy (0.233), tongue cancer (0.165), age (0.105) and present teeth (0.103) with an AUC of 0.618 [95% CI: 0.405–0.826]. The class imbalance ratio was 0.453:0.547 (MTP < 20 kPa: MTP ≥ 20 kPa).

## 4. Discussion

There are many works of literature investigating risk factors for diminished tongue pressure among dentate or elderly individuals using conventional statistical analysis, however, influencing factors such as the tumor site, defect sites, or reconstruction type on tongue pressure among individuals with head and neck tumors need to be further cross validated by establishing multiple machine learning algorithms with good predictive performances [6,13,14]. In this study, we established four machine learning predictive models and incorporated the variables shown in Table 1. According to the model performance metrics (Table 2), LR was found to outperform other models on the testing data, achieving an AUC of 0.626 [95% confidence interval (CI): 0.409–0.806] (Figure 2). In this study, variables such as glossectomy (*p* = 0.039 *), the presence of functional teeth (*p* = 0.043 *), and age (*p* = 0.044 *) were identified as significant predictors of maximum tongue pressure (Table 3).

LR shared the top spot for accuracy (0.630), F1 score (0.688), recall (0.786), and AUC (0.626), suggesting it is best at identifying true positives, making correct overall predictions, and achieving balance between precision and recall, highlighting its ability to distinguish between classes more effectively at a variety of thresholds and identify the most effective models for the given dataset and problems. Although RF, SVM, and XGB generally perform better in handling complex datasets and uncovering nonlinear relationships, LR still has its unique advantages in aspects like the linear separability of data, simplicity of the model, training speed, and probabilistic interpretation [16,17]. The selection of a suitable model is predicated on the specific requirements of the task at hand, the characteristics of the data involved, and how crucial it is to understand the model’s workings [16,17]. In practical applications, it is recommended to try multiple models and use methods such as cross validation to determine which model is most suitable for one’s specific problem. In LR, the observed discrepancy between precision and recall might be explained by multicollinearity or the small sample size. The accuracy of the four models is less than 0.8, indicating the inadequate sample size, further research with larger samples is needed to verify the results (see Section 2.5 for details).

Regarding clinical implications, in elderly patients with head and neck tumors, tongue pressure is increasingly crucial for oral functions [9] and the four models gave results consistent with those of previous studies [6,7,25,31], further validating risk factors for diminished tongue pressure and highlighting the specificity of tongue pressure in patients with head and neck tumors, indicating that machine learning models can serve as a valuable reference, even with a small sample size. Dentists, particularly maxillofacial prosthodontists, should emphasize the early identification of tongue tumors and their recurrence, even when defects are small; moreover, the timely detection of a decline in oral performance and physiological capabilities, followed by appropriate interventions such as isometric exercises and suprahyoid-targeted muscle training to strengthen tongue pressure [25,32], could play a significant role in preventing the degradation of tongue pressure. This proactive approach not only helps in maintaining the effectiveness of the swallowing mechanism, but also contributes to the overall quality of life by preventing complications associated with decreased tongue pressure as well as necessitates a coordinated effort with surgeons to ensure proper occlusion and sufficient space for dentures, thereby preserving functional teeth. Additionally, during clinical consultations, prosthodontists need to notice the specificity of maxillofacial defects in patients who are aged 65 or older, especially those who have undergone glossectomy surgery, about their swallowing function and offer specific nutritional diet guidance or use specialized appliance such as a palatal augmentation prosthesis to aid in rehabilitation [33]. Thus, tongue function should be assessed in routine visits, and the patients’ denture fit should be adjusted in a timely manner.

The average age of patients in this study was 71.98 ± 6.32 years and the MTP was 21.7 kPa, which is lower than the MTP of 26.22 kPa among people in their 70s in the general population (measured using the wireless tongue pressure measurement device) and 25.9 kPa in maxillectomy patients reported in previous studies [7,34]. Age-related declines in tongue pressure were also confirmed in the present study. The reduction in tongue pressure in elderly individuals is intricately related to several age-related conditions, including hypofunction, sarcopenia, and sarcopenic dysphagia, which ultimately lead to frailty [5]. This decline significantly impacts their ability to ingest food, contributing to malnutrition and an insufficient nutritional intake, exacerbating their health issues [35]. Research has revealed that the decrease in tongue pressure as an aging process can largely be traced back to a reduction in muscle strength [35]. This weakening of muscles is influenced by a decrease in muscle mass and the declining efficiency of the nervous system [25]. As individuals age, the nervous system’s performance diminishes, evidenced by the gradual reduction in motor unit numbers, which is particularly noticeable after the age of 60 [6,25]. Additionally, compared to their younger counterparts, older adults experience a marked decrease in the cross-sectional area of the geniohyoid muscle, which is vital for the swallowing process [25]. There is also notable atrophy in the suprahyoid muscles and an increased accumulation of fat within these muscles [25]. The increase in visceral fat deposition in older adults further contributes to tongue enlargement, compounding the swallowing difficulties [25]. Moreover, aging leads to atrophy in type 2 fibers (fast-twitch fibers), which constitute nearly 60% of the suprahyoid muscles, further diminishing muscle strength and functionality [36]. Inflammatory responses in the body also play a significant role in this context. Studies have underscored the association between inflammatory cytokines, such as interleukin-6 (IL-6) and tumor necrosis factor-alpha (TNF-α), with muscle mass and strength deterioration. It has been observed that monocytes in older individuals produce elevated levels of IL-1, IL-6, and TNF-α compared to younger individuals, highlighting an age-related increase in inflammatory activity [35]. This persistent, low-grade inflammation associated with aging, termed “inflammaging”, contributes further to the decline in muscle function and strength, thereby affecting tongue pressure and, consequently, the overall health and well-being of elderly individuals [35]. This comprehensive understanding underscores the importance of early detection and intervention to prevent the cascading effects of aging on oral and muscular health.

It has been demonstrated that a younger age and better occlusal status compensate for weaker MTP, while the loss of occlusal units and wearing a removable partial denture reduce MTP [7,8,12,31,35]. In contrast, fixed prostheses such as a bridge or implant are reported to be effective in rehabilitating and preventing decreased tongue pressure [14]. The mean value of natural teeth occlusal units in this study was 5.8, with the mean MTP being 21.7 kPa. This is low compared with the MTP of 26.4 kPa in patients with a mean number of occlusal units of 5.8 reported by Fujikawa et al. [7]. Some of the literature attributed the loss of tongue pressure in cases with fewer occlusal units to the loss of stereognosis ability by the central nervous system, which also controls masticatory rhythm [12]. Other researchers consider that the loss of muscle strength and occlusal function caused by tooth loss contributes to decreased tongue force [8,35]. Nonetheless, these studies found no positive or negative association between tongue pressure and the number of remaining teeth, which was expected to strengthen tongue pressure as compensation for tooth loss in order to maintain masticatory function [37]. Achieving optimal occlusal stability is crucial for ensuring safe swallowing [31]. This stability can be attained by preserving a higher number of teeth, ensuring more extensive occlusal contact, and increasing the support area. During swallowing, the mandible remains stationary, while the hyoid bone is elevated anteriorly by the muscles connected to the mandible; concurrently, the tongue is pressed upward against the palate. This orchestrated muscle movement during swallowing is fundamental to understanding the correlation between tongue pressure and dental health, including the number of teeth an individual has, as well as their overall frailty. This association highlights the intricate interplay between dental health and the muscular dynamics involved in the swallowing process, underscoring the importance of maintaining oral health for functional swallowing and overall well-being in individuals, especially as they age. Although the theories are contradictory, it has been confirmed that the number of functional teeth and the number of occlusal units play a crucial role, even in patients rehabilitated with a maxillofacial prosthesis. Further studies are needed to validate the relationships among occlusal condition, masticatory rhythm, and tongue pressure.

Compared with the surgical treatment of mandibulectomy and maxillectomy, glossectomy was demonstrated to be a more important predictor of tongue pressure. This is consistent with the findings of Hasegawa et al. and Hamahata et al., who reported that diminished tongue pressure was correlated with tongue cancer, suggesting that the suprahyoid and tongue muscles were dominantly involved in tongue pressure generation [10,38]. With respect to a maxillectomy and mandibulectomy, mandibular and palate support serve as two anchors for tongue pressure generation. Therefore, the hard palate not only supports the obturator, but also serves as a resistant anchor for tongue pressure [7]. However, there are discrepancies regarding how defects are associated with MTP. Fujikawa et al. noted that tongue function deteriorates after oral oncological treatment due to a loss of tissue support or complications [7,10]. Conversely, de Groot et al. did not find any impairment of tongue pressure after treatment for maxillary tumors due to the lack of tongue involvement [12]. From the perspective of a compensatory mechanism in complete denture and obturator wearers, tongue pressure is involved in not only mixing or propulsion, but also comminution and denture retention by fulfilling the role of natural teeth, in turn leading to increased tongue pressure [6,7]. For patients who underwent ablative surgery in the maxillofacial area and have poor denture stability supported by a movable flap or skin graft reconstruction, adequate bite and tongue pressure are vital for denture dexterity and oral function [12]. For larger defects, there is less support for the obturator and lower retention [7], and there is a need to determine whether a higher tongue pressure can be achieved to maintain denture retention and stability through frequent use or whether reduced tongue pressure will result from the loss of muscle mass associated with the size and location of the defect.

Regarding the limitations of the present study, despite its small sample size and the potential confounding variables not accounted for in the analysis, it is crucial to review their treatment background, which might encompass chemotherapy, radiotherapy, and neck surgery, as well as their socioeconomic status, given that prior studies have established a correlation between lower socioeconomic levels and impacts on occlusal conditions and MTP [31]. The categorization of tongue pressure was not differentiated by the type of partial dentures—fixed or removable—which might affect the restoration of tongue pressure, indicating a need for studies that analyze the retention types of dentures separately [8,14]. The tongue pressure assessment requires the fixation of anterior teeth, and the balloon should be placed at the center of the tongue. However, some participants had anterior teeth loss and subtotal glossectomy, so the balloon in those cases was positioned according to their preference, underlying the specificity of head and neck tumor patients in tongue pressure measurement. Further investigations focusing on standardized measurement methods are needed for these patients. The adaptation period for dentures may influence tongue pressure, as the muscular retention of these devices offers significant training for the perioral muscles and the tongue [6]. It is essential to conduct further studies to determine the denture adaptation period, especially for participants with specific maxillofacial defects, to ensure clarity and precision. In addition, this study had a retrospective design lacking longitudinal observations.

Future research endeavors could significantly benefit from conducting longitudinal studies that utilize extensive datasets encompassing a diverse range of ages, various types of dental retention, and a broader scope of factors including the implications of adjuvant therapy complications. Such comprehensive analyses would pave the way for establishing clearer causal or temporal relationships, thereby enhancing the accuracy and applicability of predictive algorithms. This, in turn, would not only refine the existing algorithms, but also extend their applicability to a wider spectrum of older patients afflicted with head and neck tumors, ensuring the validation of findings across a multitude of models. With the continuous evolution of automated machine learning techniques coupled with the increasingly widespread use of electronic medical records, there is a growing anticipation that machine learning and other artificial intelligence technologies will gain even greater relevance and utility in the field of oral function assessment and intervention. This progression is to promote advancements in dental care and oral health management, heralding a new era where technology-driven solutions can address complex oral health challenges with high precision and efficiency. Furthermore, the integration of manual classification outcomes obtained from an expanded cohort of dental professionals could significantly enhance the process of verifying the predictive outcomes derived from machine learning algorithms. Such a collaborative and multidisciplinary approach would not only augment the robustness of predictive models, but also foster a more holistic understanding of oral health dynamics, ultimately contributing to the development of more effective and personalized dental care strategies for the aging population. This blend of human expertise and artificial intelligence holds the promise of transforming the landscape of dental healthcare, making it more adaptive, responsive, and tailored to the unique needs of individuals.

## 5. Conclusions

Bearing in mind the limitations of this study, we can state the following conclusions:

1. In patients with head and neck tumors aged 65 years or older, the MTP was significantly influenced by factors such as glossectomy, functional teeth, and age, according to the LR model.
2. The LR model demonstrated a superior performance relative to the other two models evaluated in a small sample size, indicating the feasibility and applicability of machine learning techniques in predicting tongue pressure outcomes.
3. The presence of natural teeth and tumor sites located in the tongue emerged as consistent factors across all four models that influenced MTP, suggesting their potential utility as an early predictive marker for diminished tongue pressure.

## Figures and Tables

**Table 1:** Patient characteristics and related variables.

Characteristic	Value
Total number of patients	80
Primary tumor site	
Maxilla (%)	29 (36)
Mandible (%)	31 (39)
Tongue (%)	20 (25)
Age (years)	71.98 ± 6.32
Sex	
Male (%)	42 (53)
Female (%)	38 (47)
Number of teeth present	17.05 ± 6.73
Occlusal units (natural teeth)	5.8 ± 3.98
Occlusal units (with denture)	12.93 ± 1.44
Functional teeth	26.58 ± 2.02
Reconstruction	
Flap reconstruction (%)	37 (46)
Bone and/or metal plate reconstruction (%)	20 (25)
Perforation in maxilla (%)	16 (20)
None (%)	7 (9)
MTP ≥ 20 kPa (%)	43 (54)
MTP < 20 kPa (%)	37 (46)

Data are given as the mean ± standard deviation or number (percentage). MTP, maximum tongue pressure.

**Table 2:** Performance metrics of four models with the testing dataset.

Model	Accuracy	F1 Score	Precision	Recall	AUC
LR	0.630 [95% confidence interval (CI): 0.370–0.778]	0.688 [95% confidence interval (CI): 0.435–0.853]	0.611 [95% confidence interval (CI): 0.313–0.801]	0.786 [95% confidence interval (CI): 0.413–0.938]	0.626 [95% confidence interval (CI): 0.409–0.806]
SVM	0.593 [95% confidence interval (CI): 0.370–0.741]	0.645 [95% confidence interval (CI): 0.400–0.811]	0.588 [95% confidence interval (CI): 0.301–0.800]	0.714 [95% confidence interval (CI): 0.385–0.889]	0.582 [95% confidence interval (CI): 0.390–0.761]
RF	0.556 [95% confidence interval (CI): 0.370–0.741]	0.571 [95% confidence interval (CI): 0.320–0.762]	0.571 [95% confidence interval (CI): 0.294–0.833]	0.571 [95% confidence interval (CI): 0.308–0.846]	0.626 [95% confidence interval (CI): 0.385–0.843]
XGB	0.630 [95% confidence interval (CI): 0.444–0.815]	0.667 [95% confidence interval (CI): 0.435–0.833]	0.625 [95% confidence interval (CI): 0.375–0.857]	0.714 [95% confidence interval (CI): 0.462–0.929]	0.618 [95% confidence interval (CI): 0.405–0.826]

LR, logistic regression; SVM, support vector machine; RF, random forest; XGB, extreme gradient boosting AUC: area under the curve; F1 score = 2/([1/Recall] + [1/Precision]); Accuracy = (TP + TN)/(TP + TN + FP + FN); Precision = TP/(TP + FP); Recall = TP/(TP + FN); FN: false negatives; FP, false positives; TN, true negatives; TP, true positives.

**Table 3:** Multivariate logistic regression analysis results for the training set.

Variables	β Coefficient	*p*-Value
Glossectomy	−2.059	0.039 *
Functional teeth	−4.251	0.043 *
Age	1.674	0.044 *
Occlusal units with denture	4.166	0.052
Occlusal units without denture	2.405	0.150
Male sex	0.731	0.221
Hard tissue reconstruction	1.174	0.252
Tongue cancer	−0.263	0.750
Soft tissue reconstruction	0.198	0.811
Presence of teeth	−0.179	0.901
Glossectomy	−0.206	0.993
Perforation	−9.306	0.994

* *p* < 0.05.

## Data Availability

Data cannot be shared publicly because the data are owned and stored by Tokyo Medical and Dental University. Data are available from the Tokyo Medical and Dental University Ethics Committee for researchers who meet the criteria for access to confidential data. Contact address: 1-5-45 Yushima, Bunkyo-Ku, Tokyo 113-8510, Japan. Telephone number: +81-3-5803-5720.
